# Salivary Vascular Endothelial Growth Factor and Epidermal Growth Factor Levels in Patients with Recurrent Aphthous Stomatitis: a Meta-Analysis

**DOI:** 10.30476/dentjods.2022.94772.1809

**Published:** 2023-09

**Authors:** Paria Motahari, Davood Fathollahzadeh, Amir Alipour

**Affiliations:** 1 Dept. of Oral and Maxillofacial Medicine, Faculty of Dentistry, Tabriz University of Medical Sciences, Tabriz, Iran; 2 Dept. of Oral Pathology, Faculty of Dentistry, Tabriz University of Medical Sciences, Tabriz, Iran; 3 Dental Student, Student Research Committee, Tabriz University of Medical Sciences, Tabriz, Iran

**Keywords:** Epidermal growth factor, Vascular endothelial growth factors, Stomatitis, Aphthous

## Abstract

Recurrent aphthous stomatitis (RAS) has been identified as a common oral lesion with an unknown pathogenesis. Various studies have been conducted to show the important role of two factors named epidermal growth factor (EGF) and vascular endothelial growth factor (VEGF) in RAS, but certain results have not been achieved. The present meta-analysis was conducted to evaluate the salivary levels of EGF and VEGF in patients with RAS. For this purpose, the related articles in the Web of Science, PubMed, Embase, ProQuest and Scopus databases until January 2022 were searched and their abstracts were studied. Google scholar and scientific information database were also searched for articles in Persian. The searches were completed by the medical subject heading terms considering "recurrent aphthous stomatitis" and "saliva" in combination with "EGF" or "VEGF" keywords. All case control studies that evaluated the salivary levels of EGF and VEGF in patients with RAS were included in this study. To evaluate statistical heterogeneity between the studies, Cochrane Q and I^2^ tests were adopted. The extracted data then were used in the analysis process based on comprehensive meta-analysis software. Originally, 619 articles were considered, of which 7 articles were selected. According to this meta-analysis, salivary EGF and VEGF levels were significantly lower in the active and remission period of RAS than in healthy individuals (*p*Value< 0.05). In addition, salivary levels of these factors were significantly lower in the active stage of RAS than in the healing phase. This review study suggests that decreasing of salivary EGF and VEGF levels have significant role in the development of RAS.

## Introduction

Recurrent aphthous stomatitis (RAS) has been introduced as one of the common oral inflammatory lesions and its prevalence is 5 to 25% [ 1 ]. The maximum period of RAS was observed in the second decade and its first onset is usually in early childhood [ 1 - 2 ]. Based on the clinical manifestations, three forms of RAS can be defined including minor, major and herpetiform. Amongst them, minor type is the most common form, affecting approximately 80% of patients [ 1 - 2 ]. The ulcer appears as oval lesions on non-keratinized mucosa such as the buccal mucosa and floor of the mouth with a cover of grayish membrane and an erythematous border [ 3 ]. Ulcers commonly heal within 10 to 14 days without scarring. The diagnosis of RAS is clinically and morphologically; it does not require histopathological examination. [ 3 ]. Possible risk factors for this disease include vitamin deficiencies, food allergies, genetic predisposition, bacterial and viral infections, hormonal disorders, systemic diseases, enhanced oxidative stress, mechanical stimulation, and stress [ 4 - 7 ].

Healthy oral mucosa reacts to external stimuli, which start by a series of healing mechanisms that involve an interaction between the immune system and mucosal tissue. However, trauma to the oral mucosa causes ulceration in patients with RAS (isomorphic phenomenon) [ 8 ]. The exact reason of aphthous ulcers remains unidentified even with significant investigation. Past studies suggested that these lesions might be developed by the activity of monocytes, neutrophils, and lymphocytes on the oral epithelium that results in the release of acute inflammatory mediators (cytokines) and formation of mucosal lesions [ 9 - 10 ].

In normal physiological states, the oral epithelium could be protected by numerous defense systems, such as salivary secretions [ 11 ]. The main role of saliva is to maintain the integrity of the oral mucous membrane. Saliva includes numerous growing elements, as epidermal growth factor (EGF) and vascular endothelial growth factor (VEGF) [ 11 ]. EGF is a protein with a molecular weight of 6-kDa. Salivary EGF is a main cellular protective agent against injury and helps maintain the integrity of the oral mucosa by regulating the proliferation, growth, and migration of epithelial cells to maintain tissue homeostasis [ 12 ]. It also causes angiogenesis and plays a key role in tissue regeneration and healing [ 12 ]. VEGF is an angiogenic cytokine involved in ulcer healing and angiogenesis. It has been shown that organ growth and cell growth depend on sufficient vascular supply and sufficient angiogenesis is a basic condition for the normal functioning of the organism [ 13 ]. Pammer *et al*. [ 14 ] showed that VEGF is secreted in saliva at adequate amount for angiogenesis. Saliva has protective effects on the oral mucosa and helps in healing the oral ulcers [ 15 ]; the contribution of VEGF in this field is not well known.

Numerous studies have been performed on the expression and salivary levels of VEGF and EGF and contradictory results have been obtained [ 16 - 20 ]. Some studies have shown an increase in VEGF expression in RAS and it has been suggested that VEGF is involved as an angiogenic element in the pathogenesis of RAS and excessive angiogenesis causes loss of epithelial cell integrity [ 16 , 20 ]. It has therefore been suggested that thalidomide inhibits angiogenesis and leads to ulcer healing [ 16 ]. However, Brozovic *et al*. [ 20 ] showed that salivary levels of VEGF were higher in the healing phase than in the active (ulcer) phase, and that levels of salivary VEGF were effective in ulcer healing. Since VEGF and EGF may maintain mucosal homeostasis and on the other hand play a key role in the development and progression of oral mucosal diseases, and given the different outcomes of salivary EGF and VEGF levels in patients with RAS, it is necessary to summarize the results that have been achieved so far. Our main purpose in this study was to evaluate systematically the data obtained from studies that have examined salivary EGF and VEGF in RAS and also the characteristics of these studies.

## Literature Review

### Search Strategies

Preferred reporting items for systematic reviews and meta-analyses [ 21 ] have been adopted in the current systematic review. The main question of this study was produced according to the patient, intervention, comparison, and outcome principles [ 22 ]. The main question for this study was "Are salivary EGF and VEGF levels different in RAS patients and healthy individuals?"

English articles were searched by a librarian (F.S) from Web of Science, PubMed, Embase, ProQuest, and Scopus databases until January 2022. Google scholar and scientific information database were also searched for articles in Persian. The free and medical subject heading terms were used in a variety of combinations for collecting data. The search keywords included "recurrent aphthous stomatitis" OR "recurrent aphthous ulcers" OR "recurrent oral ulcers" AND "epidermal growth factor" OR "EGF" OR "VEGF" OR "vascular endothelial growth factor" AND "saliva" OR "salivary". 

### Inclusion and Exclusion Criteria

The studies that evaluated salivary levels of EGF and VEGF in patients with RAS were selected without restrictions of variants of RAS. For the studies selection, all titles and abstracts that were in English or Persian were reviewed, and were screened for relevance. Studies were excluded if they were non-English or non-Persian language studies, review, case report, and letters to editor or animal studies. In addition, studies involving participants with systemic disease and studies that evaluate the role of different drugs on the RAS were excluded. 

### Study Selection

After searching the articles, they were screened by two experts in three steps. In the first stage, titles and abstracts were reviewed based on the selection criteria by two independent reviewers (P.M and A.A). Disagreements were resolved by discussion with the third author (D.F). In the next step, the full text of the selected articles was studied.

### Quality Assessment

The evaluation checklist of Joanna Briggs institute was used to appraise the selected articles; thus, the risk of bias of studies was evaluated [ 23 ]. This checklist has nine criteria. Each item was answered as "yes", "no", "unclear", or "not applicable". With 1-3 "yes" scores, the risk of bias was classified as high risk and excluded from the study, 4-6 "yes" scores were rated as moderate risk, and 7-9 "yes" scores were considered as low risk. Microsoft excel software was used to organize the extracted data from each study. 

### Statistical Analysis

The extracted information included the first author/year, country, and sample size, type of disease, results, and quality score of studies. I^2^ statistics and the Cochrane test were used to assess heterogeneity between studies. These statistics represent the variation percentage between trials. As per the fixed effect model, an I^2^ of < 50% and a *p*> 0.1 suggested no substantial heterogeneity between trials. Comprehensive meta-analysis software v.2.0 was implemented to conduct the analyses. *p*< 0.05 was considered statistically significant. 

### Study Selection

**Figure 1:**
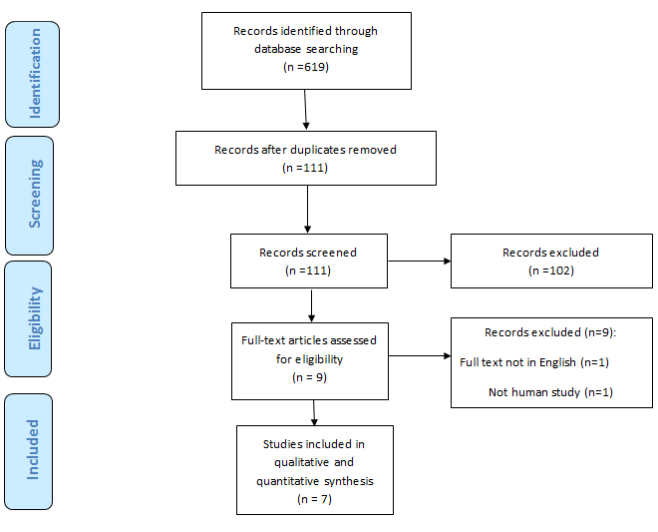
The study selection flowchart

After a systematic search of different databases, 619 articles were detected. 508 articles were excluded due to duplicate, and 102 articles were omitted following studying of the title and the abstracts. After checking the full text of the remaining articles, 7 articles were selected to be included in this meta-analysis. The flow chart of selection process is presented in Figure 1. The data of the used studies are provided in Table 1 [ 17 - 20 , 24 - 26 ].

**Table 1:** A summary of the information extracted from the studies included in this review

Authors/ year	Country	Case group (m/f)	Mean age of case group	Control group (m/f)	Mean age in control group	Type of study	Type of RAS	Factor studied	Results			Quality score
									Active stage of RAS vs. control	Remission stage of RAS vs. control	Active stage of RAS vs. remission stage of RAS	
Rezaei *et al*. [ 24 ], 2019	Iran	30	-	30	-	Case-control	Minor	EGF	No significant difference	No significant difference	No significant difference	7
Ramezani *et al*. [ 25 ], 2015	Iran	18 (7/11)	29±11.37	18 (8/10)	35± 12.84	Case-control	Minor	EGF	No significant difference	No significant difference	No significant difference	9
Adisen *et al*. [ 18 ], 2008	Turkey	16 (4/12)	29.4±8.0	60 (29/31)	32.8±7.6	Case-control	Minor	EGF	Salivary levels of EGF in active stages of RAS were lower than in control group	Salivary levels of EGF in remission stages of RAS were lower than in control group	No significant difference	8
Wu-wang/*et al*. [ 17 ], 1995	USA	28 (12/16)	20-55yr	12 (6/6)	24-45 yr	Case-control	Minor	EGF	Salivary levels of EGF in active stage of RAS were lower than in control group	Salivary levels of EGF in remission stage of RAS were lower than in control group	Salivary levels of EGF in active stage of RAS were lower than in remission stage	7
Seifi *et al*. [ 26 ], 2012	Iran	18 (5/13)	31.5± 10.7	18(5/13)	31.5± 10.7	Case-control	Minor	VEGF	Salivary VEGF levels in active stage of RAS were lower than in control group	Salivary VEGF levels in remission stage of RAS were lower than in control group	No significant difference	9
Agha-Hosseini *et al*. [ 19 ], 2005	Iran	31 (12/19)	32.16± 15.04	-	-	Case-crossover	25 minor/6 major	VEGF	-	-	No significant difference in major RAS/ in minor RAS salivary VEGF levels in active stage were lower than in remission stage	7
Brozovic *et al*. [ 20 ], 2002	Croatia	30 (14/16)	44.15± 15.53	27 (14/13)	42.04± 13.05	Case-control	20 minor /10 major	VEGF	No significant difference in minor RAS/ in major RAS salivary VEGF levels in active stage were lower than in healthy controls	No significant difference in minor and major RAS	No significant difference in minor RAS/ in major RAS salivary VEGF levels in active stage were lower than in remission stage	9

### Meta-analysis Reports of Salivary EGF

**Figure 2:**
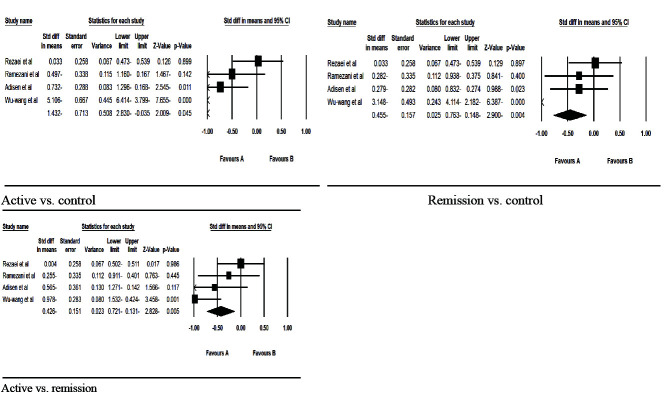
Forest diagram related to the comparison of salivary epidermal growth factor (EGF) in the control group and the active and remission stages of patients with minor recurrent aphthous stomatitis (RAS)

Figure 2 shows the forest diagrams of the meta-analysis of salivary EGF. In the 4 studies, salivary EGF was examined. In the RAS and control groups, 92 and 120 patients were studied, respectively. For patients with RAS, salivary EGF was evaluated in two stages of active and remission of ulcers. Minor RAS was considered in all studies. Heterogeneity between studies was significant (*p*< 0.05). The random-effects model was used to combine the results. A meta-analysis was performed to compare the active versus control, remission versus control, and active versus remission stages.

In active versus control comparison, patients with RAS in active stage had decreased salivary EGF values when compared to healthy controls (*p*< 0.05). In remission versus control comparison, salivary levels of EGF in the remission stage of RAS were lower than in the control group (*p*< 0.05). In active versus remission comparison, patients with RAS in active stage had decreased salivary EGF values when compared to remission stage of RAS (*p*< 0.05).

### Meta-analysis Reports of Salivary VEGF

**Figure 3:**
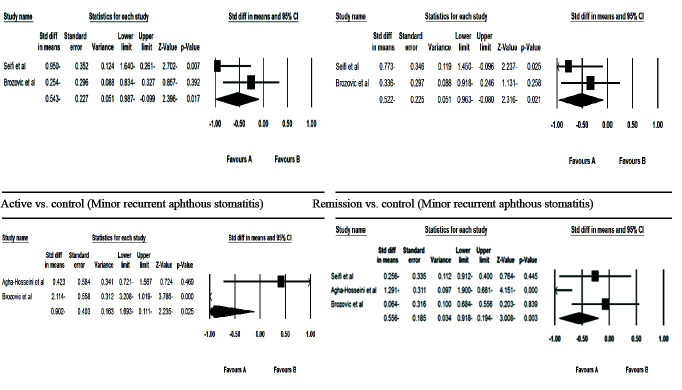
Forest diagram related to the comparison of salivary vascular endothelial growth factor (VEGF) in the control group and the active and remission stages of patients with recurrent aphthous stomatitis (RAS)

Figure 3 shows the forest diagrams of the meta-analysis of salivary VEGF. In 3 studies, salivary VEGF was examined. In the RAS and control groups, 79 and 45 patients were studied, respectively. In patients with RAS, salivary VEGF was evaluated in the two stages of active and remission of ulcers. Minor RAS was considered in all studies and major RAS was considered in only two studies. Heterogeneity between studies was significant (*p*< 0.05). The random-effects model was used to combine the results. In the case of minor RAS, a comparison was made between the active phase and the remission phase and healthy control, as well as between the remission phase and healthy controls, but in the case of major RAS, comparison of VEGF was possible only in the active phase with the remission phase.

In active versus control comparison, patients with RAS in active stage had decreased salivary VEGF values when compared to healthy controls (*p*< 0.05). In remission versus control comparison, salivary VEGF levels in the remission stage of RAS were lower than in the healthy control group (*p*< 0.05).In active versus remission comparison, patients with minor and major RAS in active stage had decreased salivary VEGF value when compared to remission stage of RAS (*p*< 0.05).

## Discussion

Our analysis showed that salivary levels of EGF in active stages of RAS were lower than in healthy individuals. The process of repairing oral tissue needs growth factors and increasing EGF production in the salivary glands in the primary stages of ulcer healing is important [ 27 ]. Salivary EGF makes the cellular mitotic response and thus is involved in RNA activation and the production of DNA, proteins, and extracellular molecules [ 28 ]. In a clinical study in a Brazilian population and according to the report of Marotta *et al*. [ 29 ], salivary EGF plays an important role in the improvement of oral lesions, periodontal disease, dry mouth, and salivary gland enlargement. Furthermore, EGF supplements are effective in ulcer healing and have positive effects of EGF in ulcer prevention [ 30 , 31 ]. Therefore, lower level of salivary EGF leads to a weakening of mucosal defense mechanisms [ 32 ]. In patients with RAS, low levels of salivary EGF may cause susceptibility to ulceration of the oral mucosa and indirectly increase antigen expression, which may stimulate or alter the immunological mechanisms [ 17 ]. Growth factors such as EGF may also have complex relationships with cytokines, and the levels of EGF may decrease because of changes in cytokine interactions in the disease. To some extent, salivary EGF levels are determined by genetic factors, as genetic factors may predispose patients to RAS [ 17 ]. This hypothesis may suggest a relationship between a deficit of this protein and the recurrence of oral ulcers in the RAS. Also decreasing of salivary EGF levels may be due to decreasing of synthesis or secretion in the salivary gland in patients with RAS [ 17 ]. In addition, abnormal salivary EGF degradation may be another explanation for the reduction in levels of EGF in RAS [ 18 ]. Wright *et al*. [ 33 ] have shown that after mucosal ulcers, new cell lines are formed that are capable of secreting EGF and promoting epithelial growth, angiogenesis, and accelerating ulcer healing [ 15 ]. In the study of Adişen *et al*. [ 18 ], it was stated that salivary EGF protects patients from injury and helps the integrity of the gastrointestinal mucosa. They showed that levels of salivary EGF were lower in remission and active stages of RAS than in healthy individuals, indicating that salivary EGF levels were reduced even in the nonappearance of oral ulcers. In a study, Wuwang *et al*. [ 17 ] reported that salivary EGF was significantly reduced in the active phase of RAS compared with the healthy controls. Overall, they showed a significant relationship between salivary EGF and RAS-induced ulcer healing. Rezaei *et al*. [ 24 ] showed that salivary EGF is not statistically significant between patients with RAS and control group. 

Interestingly, we showed salivary level of EGF in the remission phase of RAS was lower than in the control group. This result is mostly significant because it shows that patients with RAS are prone to decrease EGF levels even if no oral lesions appear. Another result was that the salivary EGF level in the active stage of RAS was significantly lower than the remission stage, which highlights the role of EGF deficiency in causing RAS. In current study, we also showed that salivary VEGF levels were lower in acute and remission stages of patients with RAS than in healthy individuals. It can be assumed that the mechanism of VEGF production in the salivary glands is disrupted in primary RAS. This can activate the self-defense mechanisms by which VEGF levels increase during the recovery period. VEGF appears to be a major mediator of angiogenesis in ulcer healing [ 12 ]. The combination of the mitogenic effect of VEGF and basal fibroblast growth factor can supplement the EGF role, leading to efficient development of new blood vessels and ulcer healing [ 34 ]. In a study by Brozovich *et al*. [ 20 ], VEGF was evaluated in patients with major and minor RAS (case group) and healthy controls. The results showed that the lower the salivary VEGF level in individuals, the higher the incidence of RAS in them [ 20 ]. Agha-Hosseini *et al*. [ 19 ], at different clinical stages of minor RAS showed significant differences in VEGF levels. Seifi *et al*. [ 26 ] in a study of salivary VEGF in patients with RAS showed that there is a statistically significant difference in VEGF levels in the clinical course of the RAS (four stages of prodromal, pre-ulcer, ulcer, and repair). The lowest amount of VEGF was seen in the ulcer stage [ 26 ]. The amount of VEGF in prodromal stage was higher than ulcer and repair but no statistically significant difference was detected in ulcer healing, pre- ulcer healing and prodromal with pre- ulcer stages [ 26 ]. They suggested that variations in VEGF levels, especially its reduction in the ulcer phase, play a key role in the pathogenesis of minor RAS development, however minor RAS is not the result of additional angiogenesis [ 26 ].

Similar to the results obtained for EGF factor, this meta-analysis showed that salivary VEGF in the active stage of RAS was significantly lower than the remission stage, which also shows the role of this factor deficiency in the development of RAS.

Concerning the limitations of our study, the sample size was limited for some groups and our search was limited to articles with English and Persian abstracts that may be considered language bias. 

## Conclusion

This review study suggests that decreasing of salivary EGF and VEGF levels may have a main role in the development of RAS. Measuring salivary VEGF and EGF levels in the future may help us identify patients at high risk for RAS.

## Conflicts of Interest

The authors declare that they have no conflict of interest.
